# Supplementary material for: Perceptions of male partners on maternal near-miss events experienced by their female partners in Rwanda
Source: PLoS One. 2023 Jun 9;18(6):e0286702. doi: 10.1371/journal.pone.0286702 (PMC10256223; doi:10.1371/journal.pone.0286702)
Supplement: S1 File — (DOCX) [file pone.0286702.s001.docx]

**Consent form for participation in the research study on Barrier challenges of utilization of maternal health services in Rwanda**

**INTRODUCTION**

Dear participant, thank you for taking part in our study on barrier challenges to maternal health in Rwanda.

The current study is designed to assess the knowledge and challenges those women of 15-49 years encounter at all levels from community to referral hospitals regarding the use and availability of maternal health services.

If you have any inquiry for the current research regarding the investigators, any ethical issues or any problem regarding the conduction of this study, please contact the chairperson of the ethical committee of the college of Medicine and health sciences on the following addresses:

Prof Kato J. NJUNWA

[njunwa@ur.ac.rw](mailto:njunwa@ur.ac.rw)

Cell phone: 0788490522

**PARTICIPATION IN THE STUDY**

Participating in this study is a full and free consent. No one will be forced to take part in the study. Apart from the researchers, no one else will have access to the data of participants; and in any case no participant’s information will be divulgated to any third party.

**RESEARCHER TEAM**

The current research on barrier challenges to maternal health is being conducted by Dr BAGAMBE Patrick, MD, MMD, PhD candidate, a senior lecturer at the University of Rwanda, obstetrics and gynecology department. This research is being supervised by Dr. Aline UMUBYEYI, MD, PhD, Dr. Laetitia NYIRAZINYOYE, PhD, lecturers at the University of Rwanda and Prof. Isaac LUGINAAH, Western University, Ontario, Canada

**WHO WILL PARTICIPATE IN THE STUDY**

Having consented to take part in the study and fulfilling the following conditions:

Women aged between 15-49 years of age and having delivered in the last year at home or in any health facility.

Having mental capacity to consent and to fully act on your own

Male partner willing to participate in the study

**WHO IS NOT ALLOWED TO TAKE PART IN THE STUDY**

Female individuals aged less than 15 or more than 49 years

Woman / partner not willing to participate in the study

**THE PROCEDURE OF THE STUDY**

The woman or her partner, will be approached by the researcher, and explained the purpose of the study, and when choosing to participate in this study, she/he will consent to participate in this study and will be given a form to fill which will not contain his/her name but his/her demographic information.

**RISKS OF PARTICIPATING IN THIS STUDY**

As the current study doesn’t involve any clinical intervention to women or their partners, there is no anticipated risk to the people participating in the study. Confidentiality is warranted during and after the study period.

**INTERESTS FOR THE PARTICIPANT**

There are no financial interests for participating in this study. As stated above, the purpose of this research is to assess the barriers to maternal health in Rwanda. Therefore, there is no direct benefit for the participants. However, we hope that by assessing the current barrier challenges to maternal health and use of maternal healthcare services will help us for future strategies in helping Rwandans and other nations how to improve the maternal healthcare delivery and thus preventing further maternal morbidity and mortality to our women.

**CONFIDENTIALITY**

As with any other type of research, the current research will not reveal any personnel detail or the disease condition of the woman/partner to anyone apart from the research team.

**PERSON OF CONTACT**

For any query about this research, please kindly contact us on the following e-mail address: [patrickgatsinzi.pg@gmail.com](mailto:patrickgatsinzi.pg@gmail.com) or the following phone number: +250 788302804

**CONSENT**

I hereby assert that I was explained thoroughly the purpose of this research, and I confirm that I was given sufficient amount of time to think about my participation in the study. I was assured about my privacy, and confidentiality was warranted by the researcher team. I confirm that I have chosen to participate in this study without financial motivation and my participation was not in any case forced by any authority, my treating doctor/ nurse or the researcher team.

Names Signature Date

…………………………………………………………………………. ………………………………….. …………………..

**AMASEZERANO Y’UBUSHAKASHATHSI**

Tunejejwe no kwemera kwanyu kwinjira muri buno bushakashatsi. Ubu bushakashatsi bugamije kumenya impamvu zitera agaore batwite n’ababyaye impamvu zituma batitabira ku gihe gahunda z’ubuvuzi bw’ababyeyi n’abana n’abana.

Niba ugize ikibazo ku bijyanye n’ubu bushakashatsi cyaba ari ikireba imyitwarire y’abakora ubu bushakashatsi, cyangwa se ikireba ukugirirwa ibanga kw’abagiye muri ubu bushakashatsi ndetse n’uburenganzira bwabo, cyangwa se ikibazo kitakunogeye ku kuba buno bushakashatsi bugiye gukorwa; niba ugize kimwe muri ibyo bibazo hamagara uhagarariye akanama gashinzwe iby’inozamigedekere y’ubushakashatsi muri kaminuza y’u Rwanda, ishami ryayo rishinzwe ubuvuzi bw’abantu kuri aderesi ikurikira:

Prof Kato J. NJUNWA

[njunwa@ur.ac.rw](mailto:njunwa@ur.ac.rw)

Cell phone: 0788490522

**IBIJYANYE NO KWINJIRA MURI BUNO BUSHAKASHATSI**

Nkuko bigenda mu bushakashatsi bwose, kwinjira muri buno bushakashatsi ni uburenganzira bwawe. Nta na rimwe byemewe kwinjira muri buno bushakashatsi ku gahato. Nta makuru akwerekeyeho ndetse n’ay’uburwayi bwawe azigera atangarizwa undi muntu uretse twebwe abashakashatsi na muganga wakuvuye, nta narimwe amazina yawe, cyangwa aderesi yawe bizigera bigaragazwa ku muntu utari muri ubu bushakashatsi.

**ABAGIYE GUKORA BUNO BUSHAKASHATSI**

Abagiye gukora buno bushakashatsi ni Dr BAGAMBE Patrick, umuganga w’indwara z’abagore n’abakobwa akaba n’umwalimu muri kaminuza y’u Rwanda. Muri buno bushakashatsi , abandi bahanga muby’ubushakashatsi bakurikira bazafatanya nawe mu rwego rw’imigendekere myiza yabwo: Dr BAGAMBE Patrick, MD, MMD, PhD candidate, a senior lecturer at the University of Rwanda, obstetrics and gynecology department. This research is being supervised by Dr. Aline UMUBYEYI, MD, PhD, Dr. Laetitia NYIRAZINYOYE, PhD, lecturers at the University of Rwanda and Prof. Isaac LUGINAAH , Western University, Ontario, Canada

**NI NDE WEMEREWE KUJYA MURI UBU BUSHAKASHATSI**

Umugore wese utwite cyangwa wabyaye kandi ari hagati y’imyaka 15-49 kandi yemera ku giti cye kujya muri ubu bushakashatsi.

Kuba ashobora kufat icyemezo ku giti cye kandi nta bumuga afite bwamubuza gufata icyemezo ku giti cye.

umufasha w’umwe mu bagore bitabiriye ubu bushakashatsi kandi yemera kujya muri buno bushakashatsi nta gahato.

**NINDE UTEMEREWE KUJYA MURI BUNO BUSHAKASHATSI**

Umugore wese uri munsi y’imyaka 15 cyangwa arengeje imyaka 49

Umugore uri kunda

Umugore urembye ku buryo imbaraga n’ubwenge bitatuma abasha kwinjira no gukomeza ubwitabire bwe muri buno bushakashatsi.

Kuba umuntu yumva adashaka kujya muri buno bushakashatsi

**UKO UBUSHAKASHATSI BUZAKORWA**

umushakashatsi hamwe n’abaganga bo mu bitaro umurwayi arimo bazabanza bamusobanurire ibijyanye na buno bushakashatsi. Abagore mu mirenge batuyemo nabo bazabazwa niba bashaka kujya mu bushakashatsi. Nyuma bazabazwa niba bemera kujya muri buno bushakashatsi noneho nibabyemera bahabwe urupapuro bagomba kudusinyiraho ko babyemeye. Umugabo we nawe igihe abyemeye azasabwa kuba yajya muri buno bushakashatsi nawe asinye abyemera.

**IBYAGO WAGIRA WINJIYE MURI BUNO BUSHAKASHATSI**

Nkuko twabisobanuye haruguru nta kintu na kimwe tuzakora k’umubiri w’umuntu uje muri buno bushakashatsi.

Amakuru yerekeye uwitabiriye ubushakashatsi nta muntu numwe utari mu bushakashatsi uzigera ayamenya kuko azakomeza kugirwa ibanga mu gihe cy’ubushakashatsi na nyuma yaho. Bityo rero nta ngaruka tubona kwinjira muri buno bushakatsi byagutera nk’umurwayi.

**INYUNGU WAKURAMO**

Ubu bushakashatsi ntago bugamije inyungu y’amafaranga. Icyo bugamije ni ukumenya impamvu zituma agagore batitabira ku gihe gahunda z’ubuzima bw’ababyeyi mu bitaro bitandukanye. Bityo rero nta nyungu yako kanya ku bazajya muri buno bushakashatsi. Gusa twizera neza ko ubu bushakashatsi buzaduha amakuru ku mpamvu zituma/zibuza abagore kwitabire serivisi z’ubuvuzi bw’ababyeyi mu Rwanda bityo tukazakuramo amakuru yakunganira Leta mu buryo izi serivisi zitangwa ku baturage.

**KUGIRIRWA IBANGA**

Nkuko twabibasonuriye, ubu bushakashatsi nta na rimwe buzagaragaza umwirondoro w’umuntu cyangwa uburwayi bwe ku wundi muntu utari muri bano bashakashatsi. Haba mu gihe ubu bushakashatsi burimo gukorwa na nyuma yaho nta na rimwe umwirondoro wawe cyangwa uburwayi bwawe buzigera bugaragarizwa undi muntu utari muri aba bashakashatsi.

**UBURYOZWE**

Gusinya cvangwa kudasinya aya masezerano ntibikuraho uburenganzira wari usanganywe bwo kuvurwa cyangwa se ngo bikongerere ubuzare. Nta nicyo bihindura ku burenganzira n’inshingano z’abashakashatsi.

**NINDE WABAZA IGIHE UGIZE IKIBAZO**

Niba ugize ikibazo kubijyanye n’ubu bushakashatsi twandikire kuri murandasi kuri aderesi ikurikira: [patrickgatsinzi.pg@gmail.com](mailto:patrickgatsinzi.pg@gmail.com) cyangwa uduhamagare kuri numero 0788302804.

**AMASEZARANO**

Ndemeza ko numvise neza icyo buno bushakashatsi bugamije, ndemeza kandi ko nabonye umwanya uhagije wo kubitekerezaho. Nizeye neza kandi ko amakuru yose anyerecyeyeho azagirwa ibanga n’abashakashatsi. Ndemera ko ninjiye muri buno bushakatsi kandi ko kwemera kujya muri ubu bushakashatsi nta nyungu y’amafaranga kandi ko nta gahato nashyizweho n’abayobozi, muganga umvura cyangwa n’abashakashatsi.

Amazina yumurwayi umukono itariki

…………………………………………………………………………….. ……………………… …………………………………
