# Supplementary material for: Perceptions of male partners on maternal near-miss events experienced by their female partners in Rwanda
Source: PLoS One. 2023 Jun 9;18(6):e0286702. doi: 10.1371/journal.pone.0286702 (PMC10256223; doi:10.1371/journal.pone.0286702)
Supplement: S2 File — (DOCX) [file pone.0286702.s002.docx]

**Interview guide form for participation in the research study on Barrier challenges of utilization of maternal health services in Rwanda**

**Interviewer:** how were you feeling when your wife was in hospital?

**Respondent:**

**Interviewer:** how were your daily activities affected when your wife was sick?

**Respondent:**

**Interviewer:** how did you know that your wife was sick?

**Respondent:**

**Interviewer:** during her operation was the uterus removed?

**Respondent:**

**Interviewer:** how did you perceive the medical care given to your wife?

**Respondent:**

**Interviewer:** what possible bad outcomes you were thinking could happen to your wife?

**Respondent:**

**Interviewer:** when your wife was sick, did it affect your family economy?

**Respondent:**

**Interviewer:** how did the sickness of your wife affect the relationship with your neighbours?

**Respondent:**

**Interviewer:** during her sickness were you able to talk to health professionals that were treating her?

**Respondent:**

**Interviewer:** how are you leaving now after her sickness?

**Respondent:**

**Interviewer:** whatever happened to your wife, did it induce any psychological trauma to you?

**Respondent:**

**Interviewer:** who do you think helped you the most when your wife was sick?

**Respondent:**

**Interviewer:** what advice would you give to husbands who have pregnant wives?

**Respondent:**

**Interviewer:** what do you think the government should do to improve the health care for pregnant women?

**Respondent:**

**Interviewer:** Is there any other question or comment you would want to share with us?

**Respondent:**

**Interviewer:** Thank you very much for your time
