# Supplementary material for: Perceptions of male partners on maternal near-miss events experienced by their female partners in Rwanda
Source: PLoS One. 2023 Jun 9;18(6):e0286702. doi: 10.1371/journal.pone.0286702 (PMC10256223; doi:10.1371/journal.pone.0286702)
Supplement: Attachment — Submitted filename: Response letter 2.docx [file pone.0286702.s003.docx]

January 10, 2022

Dr Patrick Gatisinzi Bagambe

Senior Lecturer of Obstetrics and Gynaecology

University of Rwanda

Dear Sir/madam, greetings

I am writing to clarify with regards to the data availability statement. During the initial submission an error was made stating that there is supporting information files. When in fact all the relevant and summary data underlying the current study findings were within the paper. And there are no other supporting information files. As per the ethics approval granted by the Research Ethics Boards of the University of Rwanda (No252/CMHS IRB/2018) and the University of Western Ontario (REB#: 111859) for the Training Support Access Model (TSAM) project, the raw data must not be hosted in a public repository. Thus, access to these raw data would require further approval from both the Research Ethics Boards of the University of Rwanda and the University of Western Ontario. For data access please contact Prof. David F. Cechetto, the Director of the TSAM project in Rwanda via his email: [cechetto@uwo.ca](mailto:cechetto@uwo.ca) or [ethics@uwo.ca](mailto:ethics@uwo.ca)

Best regards
