# Supplementary material for: Perceptions of male partners on maternal near-miss events experienced by their female partners in Rwanda
Source: PLoS One. 2023 Jun 9;18(6):e0286702. doi: 10.1371/journal.pone.0286702 (PMC10256223; doi:10.1371/journal.pone.0286702)
Supplement: Attachment — Submitted filename: Response to Reviewers (4).docx [file pone.0286702.s004.docx]

Patrick G Bagambe

Senior Lecturer of Obstetrics and Gynecology

University of Rwanda

patrickgatsinzi.pg@gmail.com

+250 788 302 804

August 23, 2022

Dear Sir/Madam

**Re: PLOS-ONE-D-21-39209: “Perceptions of male partners on maternal near-miss events experienced by their female partners in Rwanda”.**

Please find enclosed a revised version of our manuscript number PLOS-ONE-D-21-39209. The Revisions are based comments and suggestions from yourself and the reviewers. The manuscript has been revised considerably. Please find enclosed a detailed response to the comments the manuscript reviewers.

The manuscript has not been submitted for consideration for publication elsewhere. There are no financial agreements or other conflicts of interest to disclose.

I look forward to your evaluation of the revised manuscript.

Sincerely,

Patrick Bagambe

**Response to Reviewers and editors' comments:**

Interview guide.

The interview guide should be described in the methods section briefly.

***The interview guide has been described in the methods section***

A description of the coding tree with theme(s) should be provided.

***The Coding tree has been described in the methodology section and the six emerging themes are documented in the results section***

The authors should state how many meaning units and codes were extracted from the data.

***64 meaning units were extracted and condensed into 25 codes. Those codes make the six broad themes. A table describing the codes and themes was added.***

In page 4, the authors state, ‘Once a woman with near-miss was identified from the TSAM registry, we checked the hospital registry to identify her local village and we used the Community Health Worker’s …….’ But in the limitations of the study we find that the interviews were conducted months after discharging the women from the hospital. For the sake of clarity, the interval between the discharge of a mother from hospital and the interview with her spouse should be included in table 1 or 2.

***The time interval from maternal discharge to the interview with the male partner was 12 to 24 months among 12 participants but some were interviewed after two years. More details were summarized in table 2.***

Please find alternative phrasing to explain 'relieved aftermath depression' (eg do you mean associated with lower levels of depression in the postpartum period? improved recovery?) and 'a realized birth expectations'.

***Alternative phrasing has been added***
